# Blister Fluid Induces MMP-9-Associated M2-Type Macrophages in Bullous Pemphigoid

**DOI:** 10.3389/fimmu.2019.01858

**Published:** 2019-08-07

**Authors:** Meriem Riani, Céline Muller, Camille Bour, Philippe Bernard, Frank Antonicelli, Sébastien Le Jan

**Affiliations:** ^1^Laboratory of Dermatology, EA7509 IRMAIC, University of Reims-Champagne-Ardenne, Reims, France; ^2^Department of Dermatology, University Hospital, University of Reims-Champagne-Ardenne, Reims, France; ^3^Department of Biological Sciences, Immunology, UFR Odontology, University of Reims-Champagne-Ardenne, Reims, France

**Keywords:** bullous pemphigoid, autoimmunity, inflammation, macrophage polarization, MMP-9

## Abstract

Bullous pemphigoid (BP) is a cutaneous autoimmune disease, characterized by an inflammatory cascade leading to blister formation. Although macrophages were shown to participate in BP pathophysiology, their role in the blister formation process still needs to be investigated. We here addressed the influence of serum and blister fluid (BF) from patients with BP on the polarization status of macrophages with regards to the metalloproteinase-9 (MMP-9) expression. We demonstrated that several markers related to the alternatively activated macrophage phenotype (M2) including IL-10, TARC, arginase, TNFα, and IL-1RA were meaningfully increased in BF of patients with BP. We further showed that BF, but not serum from patients with BP, significantly induced the expression of CD163, CD206, and IL-10 in BP monocyte-derived macrophages (MDMs). Notably IL-10 was the only cytokine to be correlated to the reference clinical score, BP disease activity index (BPDAI), especially to the inflammatory BPDAI subscore evaluating urticarial and erythematous skin lesions (*r* = 0.57, *p* = 0.0004). We also found elevated levels of MMP-9 to M2-type macrophages *ex vivo* and highlighted the presence of CD163^+^ MMP-9^+^ macrophages histologically, at skin lesional site. Finally, we showed that methylprednisolone reduced MMP-9 levels in MDMs without modifying the other M2 markers. All together these results strongly support the presence of M2-phenotype macrophages with pro-inflammatory properties susceptible to favor blister formation in BP.

## Introduction

Bullous pemphigoid (BP) is the most common skin autoimmune subepidermal blistering disease (1, 2). The disease typically presents in the elderly with a generalized pruriginous, erythematous and bullous eruption. Biologically, BP is characterized by the binding of autoantibodies directed against two components of the hemidesmosome, BP230 and BP180 that generates an inflammatory response critical for blister formation. A pathophysiological mechanism of blister formation was established, which typically involved a variety of cellular types including eosinophils, neutrophils, lymphocytes, mast cells, and macrophages (3, 4). Mast cell activation upon immune complex binding play a major role in neutrophils recruitment, while macrophages were supposed to rather amplify the neutrophil infiltration in a mast cell-dependent fashion (5, 6). However, the role of macrophage polarization has not been taken into consideration in the pathophysiological BP process, and especially regarding the production of MMP-9, a metalloproteinase widely involved in blister formation (7).

Macrophages display a remarkable plasticity and can change their physiology in response to environmental factors with at one extremity the inflammatory or classically activated macrophage M1 phenotype and at the other, the anti-inflammatory or alternatively activated macrophage M2 phenotype (8). The M1 phenotype is classically induced by microbial products or pro-inflammatory cytokines such as IFN-γ and TNFα, whereas molecules such as IL-4, IL-13, M-CSF, immune complexes, IL-10, and glucocorticoids favor the orientation toward the M2-type macrophage phenotype. In addition, M1-type macrophages have been shown to produce high levels of pro-inflammatory molecules such as TNFα, IL-1, IL-6, IL-23, IL-12, type-I IFN, reactive nitrogen intermediate (RNI), reactive oxygen intermediate (ROI), CXCL9, CXCL10, and CXCL11 (9), whereas M2 macrophages express IL-4, IL-10, CD163, and CD206 and promote tissue regeneration and repair (10, 11). Hence, a switch in macrophage phenotype could be of importance in the pathogenesis of autoimmune and inflammatory diseases (12–14).

In line with previous reports on macrophage polarization (15, 16), the presence of immune complexes at the skin lesional site in BP should theorically favor the presence of M2 type macrophages. However, we recently showed that BP was associated with the production of IL-23 and CXCL10, two markers of the M1 macrophage phenotype (17–19), thus further questioning on the status and the role of macrophages in the early, inflammatory process leading to blister formation in BP.

We here aimed at understanding the influence of serum and blister fluids (BF) of patients with BP on macrophage polarization by analyzing the expression of several M1/M2 markers as well as the expression of MMP-9. As corticosteroids, the main treatment of BP, favor M2-macrophage polarization, we also investigated the effect of methylprednisolone on the expression of these macrophage markers and MMP-9.

## Methods

### Patients and Study Design

This ancillary study is part of a main prospective, single-center study that was conducted in the Department of Dermatology at Reims University Hospital (French Referral Center for Autoimmune Bullous Diseases) between September 2013 and July 2017. Consecutive patients with newly diagnosed BP were included using the following criteria: clinical features typical of BP with presence of at least three out of four well-established criteria by Vaillant et al. (20); subepidermal blister on skin biopsy; and deposits of IgG and/or C3 in a linear pattern along the epidermal basement membrane zone by direct IF. Sera and BFs from patients were collected at time of diagnosis (V1). Serum samples from age- and sex-matched patients without inflammatory and autoimmune diseases admitted to the trauma department of the Reims University Hospital were used as controls. The investigation was conducted under the approval of the Ethic Committee of the University Hospital of Reims (CNIL authorization DR-2013-320), and all of the subjects gave their informed and written consent before participating in the study in accordance with the Helsinki Declaration.

### Cell Preparation

THP-1 monocytic cell line, kindly provided by Dr. S. Hart from University of Edinburgh, UK, was cultured in RPMI 1640 medium (Life Technologies), supplemented with 10% fetal bovine serum (FBS), 2 mM glutamine, 25 U/ml penicillin, and 25 U/ml streptomycin at 37°C in a humidified 5% CO_2_ incubator. THP-1-derived macrophages were generated as described previously (21). Briefly THP-1 cells were seeded at a concentration of 2 × 10^6^ cells per milliliter in FBS-free medium and treated with 320 nM PMA for 24 h. To generate M1-polarized THP-1 macrophages, THP-1 cells were treated with 320 nM PMA for 6 h and then cultured with PMA plus 20 ng/ml IFN-γ and 20 ng/ml TNFα for 18 h. To generate M2-polarized THP-1 macrophages, THP-1 cells were treated with 320 nM PMA for 6 h, and then cultured with PMA plus 20 ng/ml IL10 for another 18 h. Cells and culture media were then harvested and analyzed for gene expression and MMP-9 secretion, respectively. For stimulation, THP-1 cells were treated with 320 nM PMA in FBS-free medium for 6 h and then cultured for 18 h with 320 nM PMA plus 10% BF or 10% serum from control subjects (CTR serum) or BP patients (BP serum). When mentioned methylprednisolone (Sigma) was introduced with serum at a concentration of 10 μM. Cells were then harvested and analyzed for gene expression.

Peripheral blood mononuclear cells (PBMCs) from control subjects or patients with BP were obtained by density-gradient centrifugation from EDTA-treated whole blood using a density gradient medium (Granulosep, Eurobio-Abcys). Monocytes were purified from PBMCs by positive selection using CD14 immunomagnetic beads (MACS; Miltenyi Biotech) according to manufacturer instructions. To generate monocyte-derived macrophages (MDMs), freshly isolated monocytes were cultured at a concentration of 2 × 10^6^ cells per milliliter for 7 days in RPMI 1640 medium (Life Technologies), 2 mM glutamine, 25 U/ml penicillin, 25 U/ml streptomycin and 10% serum from control subjects or BP patients. Culture media was replaced at day 4. When stimulated, MDM were treated for 24 h with 10% BF simultaneously or not with methylprednisolone. Cells were then harvested and analyzed for gene expression.

### Gene Expression Analysis

Total RNA from isolated and cultured cells was extracted using TRI-Reagent (Euromedex). Reverse transcription was performed from 1 μg of total RNA using Maxima First Strand cDNA kit with dsDNAse (Life Technologies) according to manufacturer's instructions. *cxcl10, il-23, inos, tnf*α*, CD163, CD206, il-10, il-1ra, and mmp9* gene expressions were analyzed by quantitative real-time PCR using Platinum SYBR Green qPCR SuperMix-UDG kit (Invitrogen) on the LightCycler system (Roche Diagnostics). Relative quantification was performed using the *GAPDH* as a reference gene.

### *In situ* Protein Expression Analysis

Perilesional skin biopsy specimens performed in patients with BP before introduction of any corticosteroid treatment were obtained from the Department of Anatomo-Pathology at Reims Hospital. Tissues were fixed in paraformaldehyde, embedded in paraffin, and sectioned. To visualize M2 type macrophages, immunostaining with a mouse monoclonal anti-human CD163 antibody (Clone MRQ26, Roche Diagnostics, Meylan, France) was performed by using routine methods with a biotinylated anti-mouse secondary antibody (BA-2000; Vector Laboratories, Burlingame, USA) and the ABC-peroxidase complex (Vector Laboratories) with diaminobenzidine-H_2_O_2_ used as the chromogen for detection. In order to identify cells positive for both CD163 and MMP-9, simultaneous staining was performed with mouse monoclonal anti-human CD163 antibody (Clone MRQ26, Roche Diagnostics, Meylan, France) and rabbit anti-human MMP-9 [MMP-9 (E11), Santa Cruz Biotechnologies] and followed by incubation with matched secondary antibodies: chicken anti-goat IgG Alexa Fluor 488 and chicken anti-mouse IgG Alexa Fluor 594, respectively (Invitrogen). Images were captured with confocal microscope (LSM 710; Zeiss) using Zen software.

### Determination of Cytokine Levels

M1 type (TNF-α, IFN-γ) and M2 type (arginase, TARC, IL-10, IL-1RA) macrophage marker concentrations in sera and BFs were determined using the bead-based immunoassays LEGENDplex *Human Macrophage/Microglia Panel* and *Human Th Cytokines Panel* (BioLegend, San Diego, CA, USA). The assays were performed in 96-well plates following the manufacturer's instructions. For measurements a LSR Fortessa flow cytometer (BD Biosciences) was employed, and data were evaluated with the LEGENDplex™ Data Analysis software.

### Zymography

The quantity of MMP-9 was determined on conditioned media by the zymography technique. Briefly, samples were electrophoresed on a 10% SDS polyacrylamide gel impregnated with 0.1% of gelatin as substrate under non-reducing conditions (22). The gels were subsequently fixed and stained with 0.25% Coomassie brilliant blue R-250 to visualize the proteolytic activity bands. Quantification was performed using the ImageJ software (National Institutes of Health, Bethesda, MD).

### Statistical Analysis

Descriptive statistics such as means and SEMs were conducted for all quantitative measures. The distribution of the variables was assessed using D'Agostino and Pearson omnibus normality test. As population could not be assumed to be normal and some of the groups examined were small, we used non-parametric testing to compare populations in this study. Comparisons between two groups were performed using the exact Wilcoxon signed-rank test for paired data and the Mann-Whitney test for unpaired data. Correlations were performed using non-parametric Spearman's correlation test. The results were considered significant if *p-*values were 0.05 or less.

## Results

### BF Is Associated With a M2 Macrophage Cytokine Profile in BP

**Figure 1:**
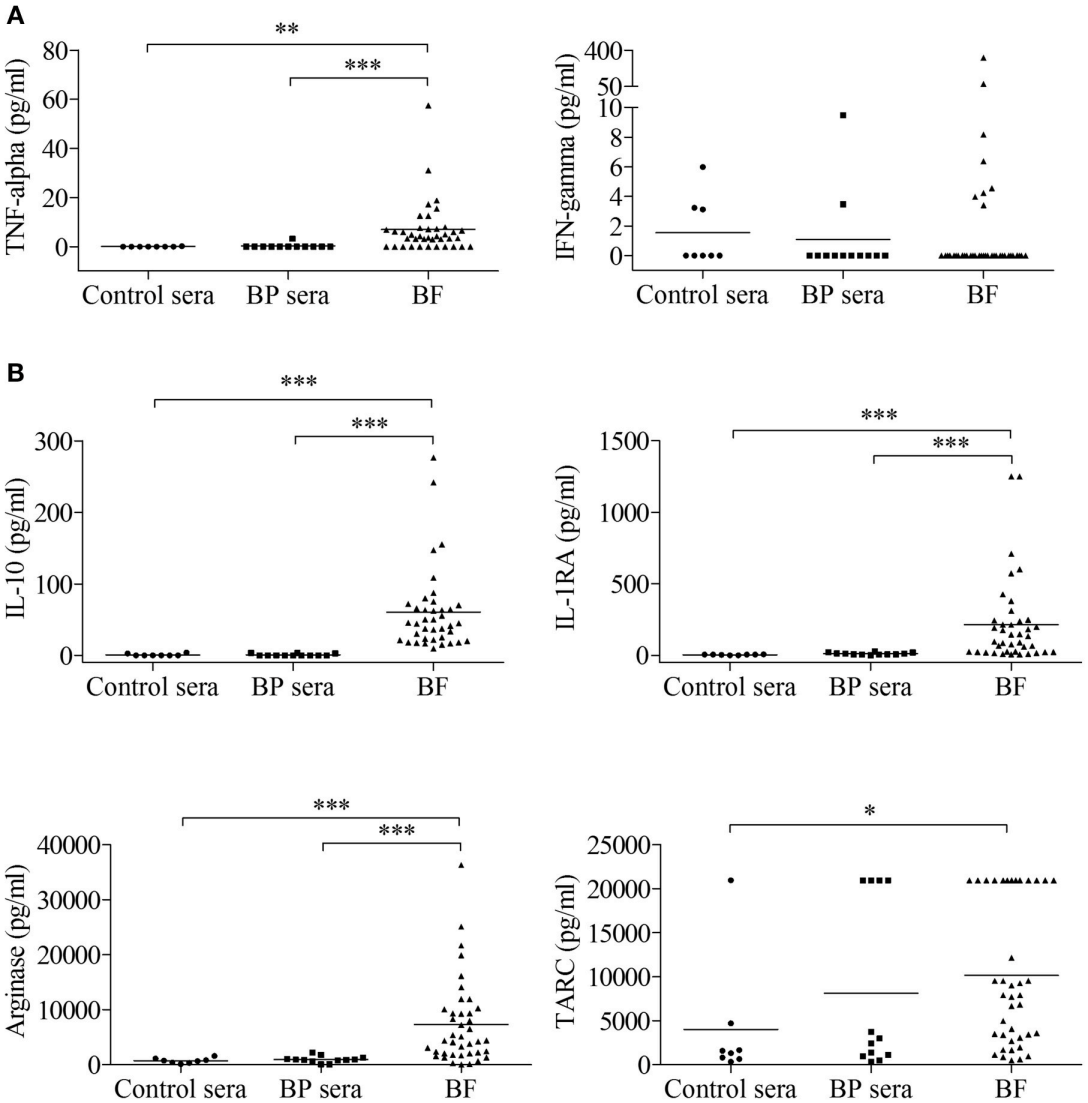
Detection of macrophage-associated cytokines in serum and blister fluid (BF) of BP patients. The concentrations of a panel of M1-type **(A)** and M2-type **(B)** macrophage markers were measured in serum from control subjects (Control sera, *n* = 8) and in serum (BP sera, *n* = 12) and BF (*n* = 40) from patients with BP at time of diagnosis using the bead-based immunoassays LEGENDplex *Human Macrophage/Microglia Panel* and *Human Th Cytokines Panel*. Non-parametric unpaired Mann–Whitney's test was used to compare populations (^*^p < 0.05; ^**^p < 0.01; ^***^p < 0.001).

First, we measured the concentration of several markers associated with macrophage polarization both in sera and BFs collected from patients with BP at time of diagnosis and before treatment and in control sera (Figure 1). The concentrations of TNF-α but not IFN-γ, both used as M1 type macrophage markers (Figure 1A), and of IL-10, IL-1RA, arginase used as M2 type macrophage markers (Figure 1B) showed significant increase in BFs compared with their concentrations in both control and BP patient sera. Concentrations of TARC, another M2 marker, were significantly increased in BF compared with control sera but not with BP sera. Notably, no variation was evidenced in the serum of BP patients with respect to control sera (Figures 1A,B).

### BF Rather Favored M2 Type Than M1 Type Macrophage Phenotype

**Figure 2:**
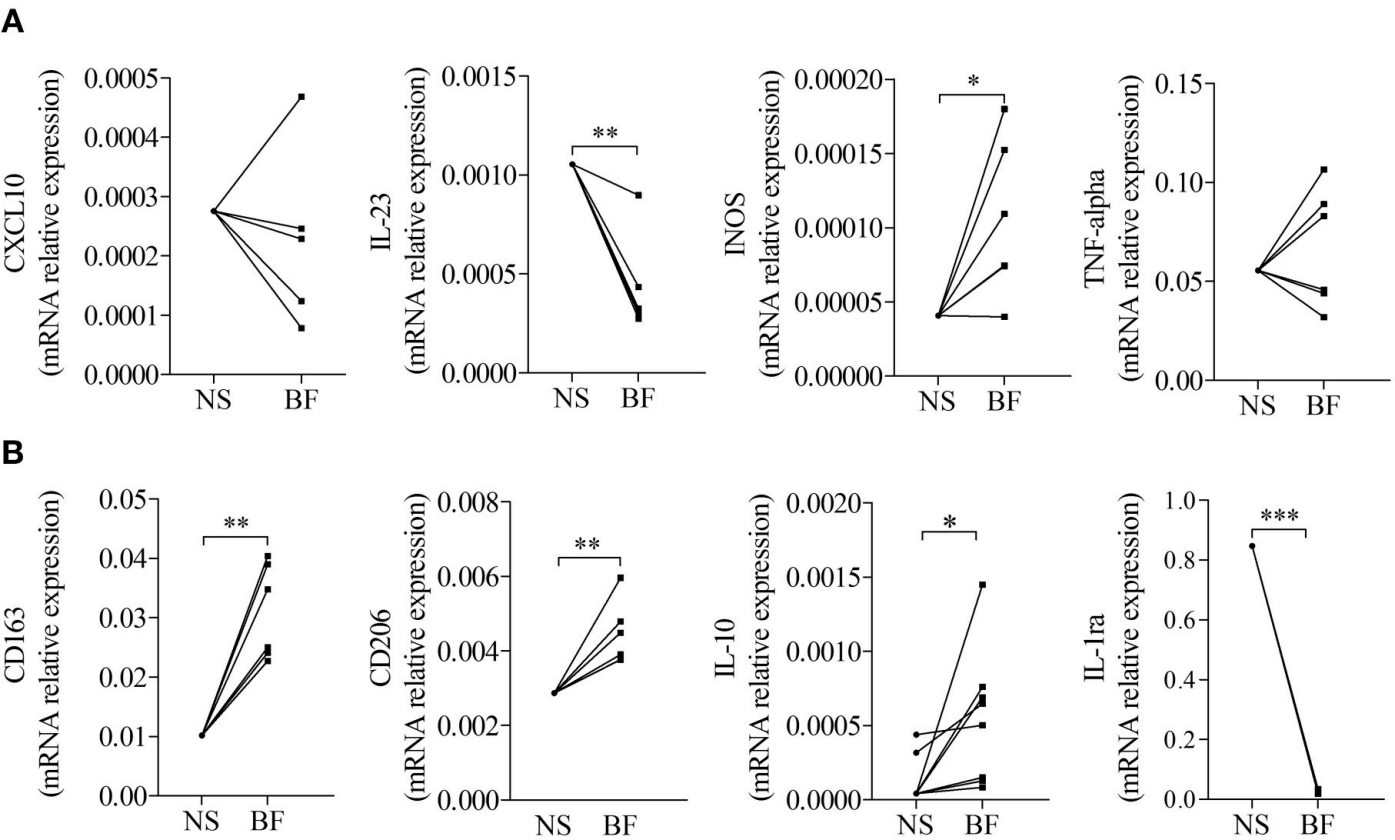
Blister fluid (BF) from patients with BP affected the expression of M1/M2-type macrophage markers in THP-1 derived macrophages. M1-type **(A)** and M2-type **(B)** macrophage marker expressions were analyzed by real-time qPCR in THP-1 derived macrophage unstimulated (NS) or stimulated with BF (BF) from patients with BP. A paired *T*-Test was performed for statistics (^*^p < 0.05; ^**^p < 0.01; ^***^p < 0.001).

**Figure 3:**
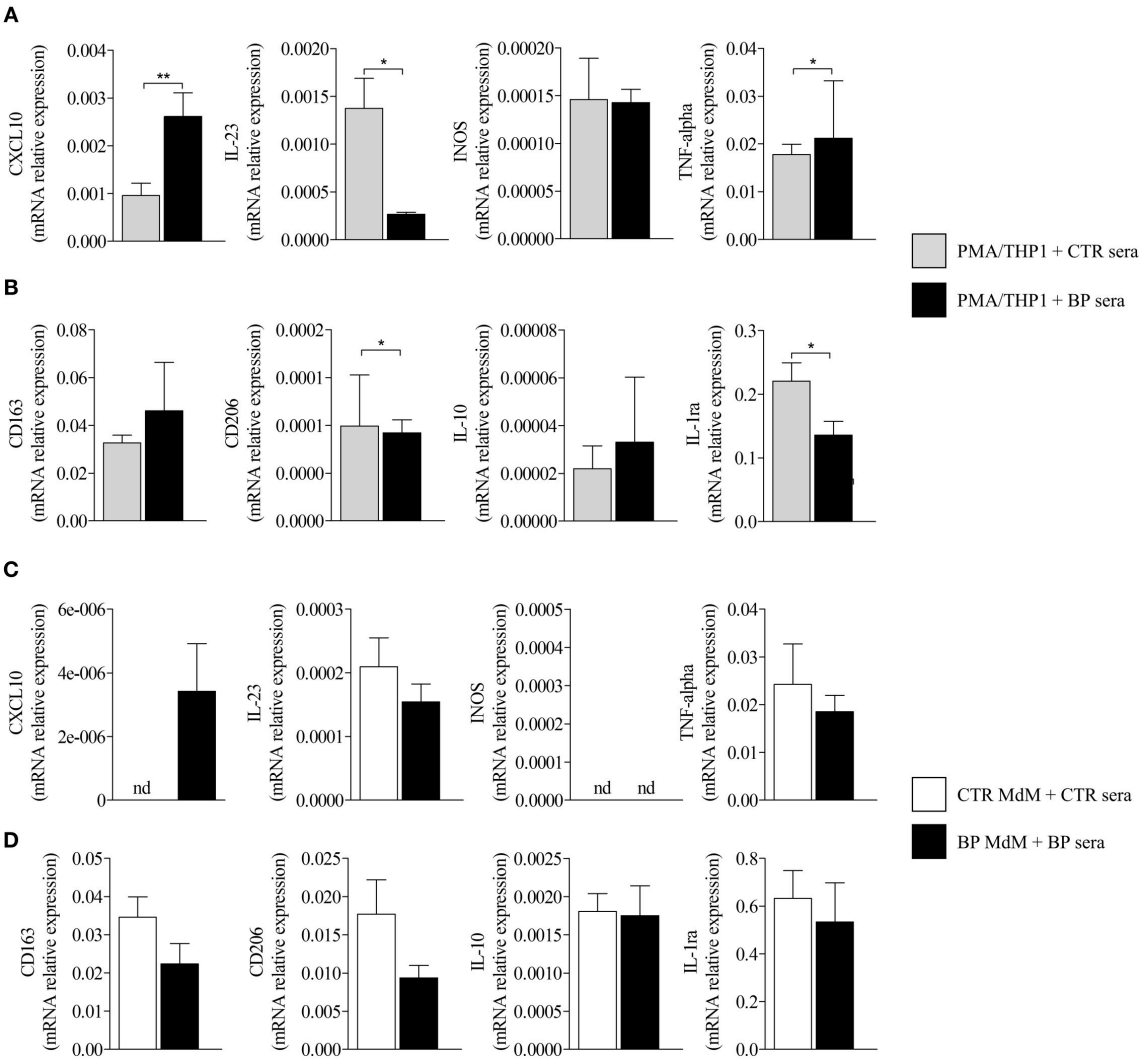
Regulation of M1-type and M2-type macrophage marker expression in THP-1- and monocyte-derived macrophages. M1-type **(A,C)** and M2-type **(B,D)** macrophage marker expressions were analyzed by real-time qPCR in CTR or BP serum-stimulated macrophages originated either from PMA-derived THP-1 cells **(A,B)** or from CTR or BP monocytes differentiated with their autologous serum for 7 days **(C,D)**. The error bars denote the mean ± SEM. Non-parametric unpaired Mann–Whitney's test was used to compare populations (^*^p < 0.05; ^**^p < 0.01; nd, not detected).

**Figure 4:**
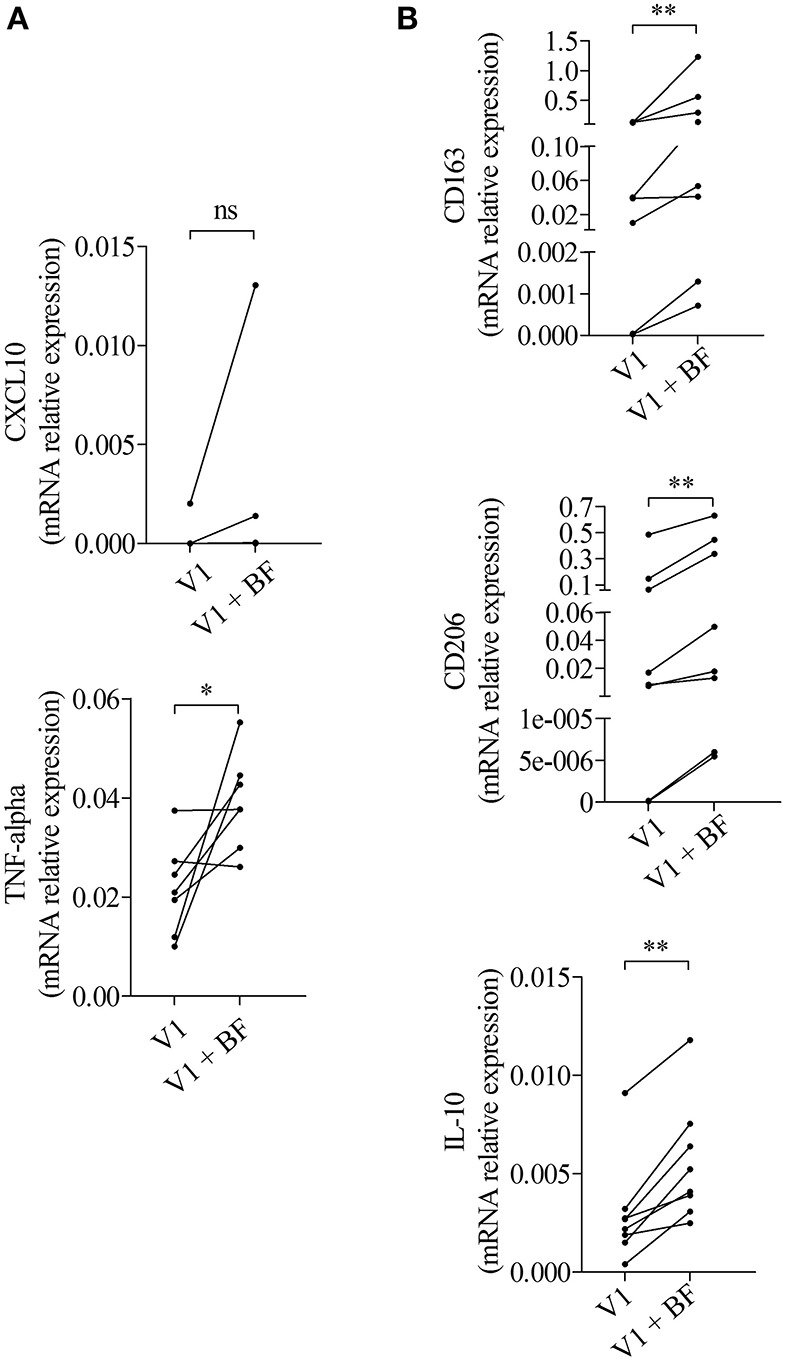
BF essentially increased the expression of M2-type macrophage markers in BP monocyte-derived macrophages. M1-type **(A)** and M2-type **(B)** macrophage marker expressions were analyzed by real-time qPCR in macrophages issued from BP monocytes differentiated for 7 days with the autologous serum collected at diagnosis (V1) and stimulated with BF for 24 h (V1 + BF). Non-parametric paired Wilcoxon's test was used to compare populations (ns, not significant; ^*^p < 0.05; ^**^p < 0.01).

To investigate whether those expressions were related to macrophages, we next tested the effects of BF from BP patients on the expression of M1 and M2 macrophage markers by the macrophages derived from PMA-induced THP-1 monocytic cells (Figure 2). Among the M1-associated markers, a significant increase in iNOS expression was observed upon BF stimulation (*p* < 0.05), whereas a decrease for IL-23 mRNA expression (*p* < 0.01) and no variation in CXCL10 and TNFα mRNA expression were evidenced (Figure 2A). Analysis of the M2-associated markers showed that BF stimulation induced an increase in CD163, CD206, and IL-10 (*p* < 0.01, *p* < 0.01, and *p* < 0.05, respectively), but a decrease in IL-1RA expression (*p* < 0.001), suggesting that BF rather favored M2 type than M1 type macrophage phenotype (Figure 2B). We also used the PMA-induced THP-1 macrophages to investigate whether BP serum could favor either an M1 or an M2 phenotype (Figures 3A,B). Compared with control serum, short term stimulation with BP serum of PMA-induced THP-1 macrophages increased CXCL10 and TNFα expression (*p* < 0.01 and *p* < 0.05, respectively) and decreased the expression of IL-23, IL-1RA, and CD206 (*p* < 0.05; Figure 3A). Such variations were not observed when macrophages were differentiated from monocytes (MdM) isolated from control or BP patients through a longer period (7 days) in presence of serum (Figures 3C,D). Based on these results, we next investigated whether the differentiation of BP monocyte into macrophage upon serum stimulation affected the effects of BF on the expression of macrophage markers (Figure 4). Stimulation of BP-MdM with BF significantly increased TNFα, CD163, CD206, and IL-10 (*p* < 0.05, *p* < 0.01, *p* < 0.01, and *p* < 0.01, respectively) but not CXCL10 expression (Figures 4A,B).

### M2 Type Macrophage Profile Was Associated With Disease Activity in BP

**Figure 5:**
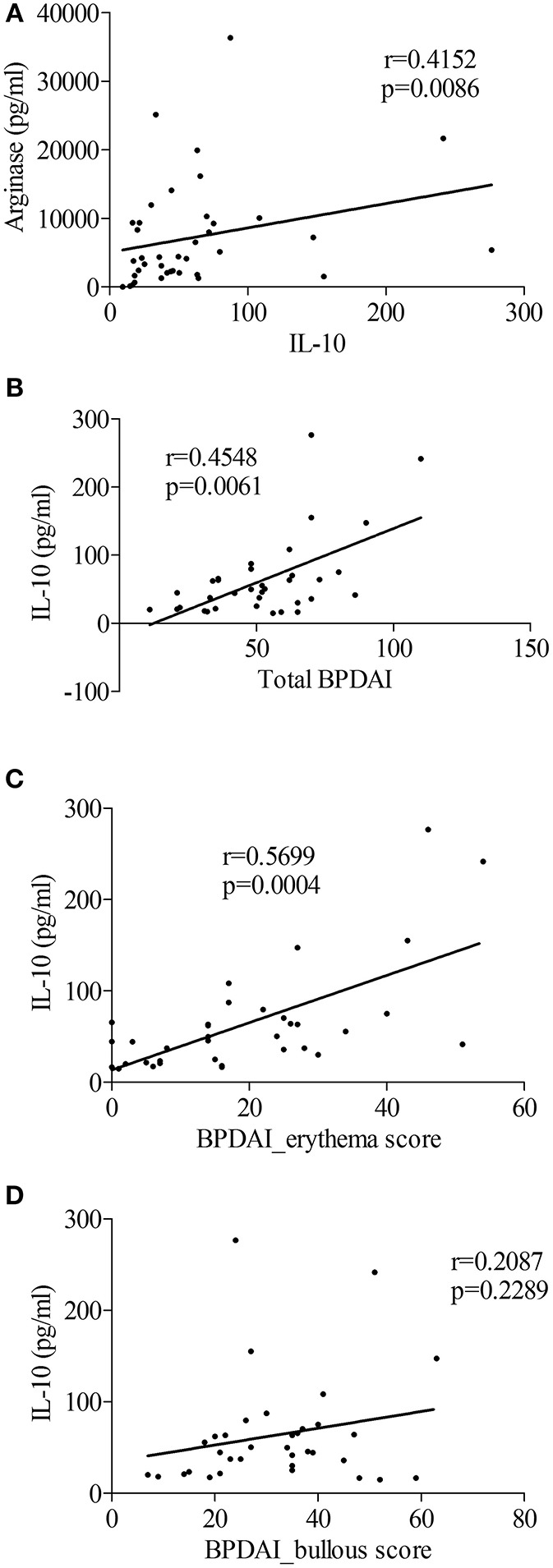
The M2-type macrophage marker IL-10 was related to BP disease activity. **(A)** Correlation between levels of IL-10 and the M2-type macrophage marker Arginase in blister fluid of patients with BP at diagnosis was analyzed using the nonparametric Spearman's correlation test. **(B–D)** Correlations between total BPDAI score **(B)**, blister/erosion **(C)**, and erythema **(D)** BPDAI subscores and IL-10 levels measured in BF were analyzed using the non-parametric Spearman's correlation test (*n* = 35).

Within the BF, IL-10 concentrations were correlated to those of arginase (Figure 5A, *r* = 0.41, *p* = 0.0086). Also, IL-10 was the only cytokine among all cytokines tested in Figure 1 to be related to the clinical activity score. Indeed IL-10 concentrations in the BF were correlated with disease activity measured by the total BPDAI score (Figure 5B, *r* = 0.45, *p* = 0.006), and especially with the inflammatory BPDAI subscore associated with urticarial and erythematous plaques (Figure 5C, *r* = 0.57, *p* = 0.0004), but not with the subscore bound to skin lesions (Figure 5D, *r* = 0.21, *p* = 0.23).

### BF Increased the Production of MMP-9 by M2 Type Macrophages in BP

**Figure 6:**
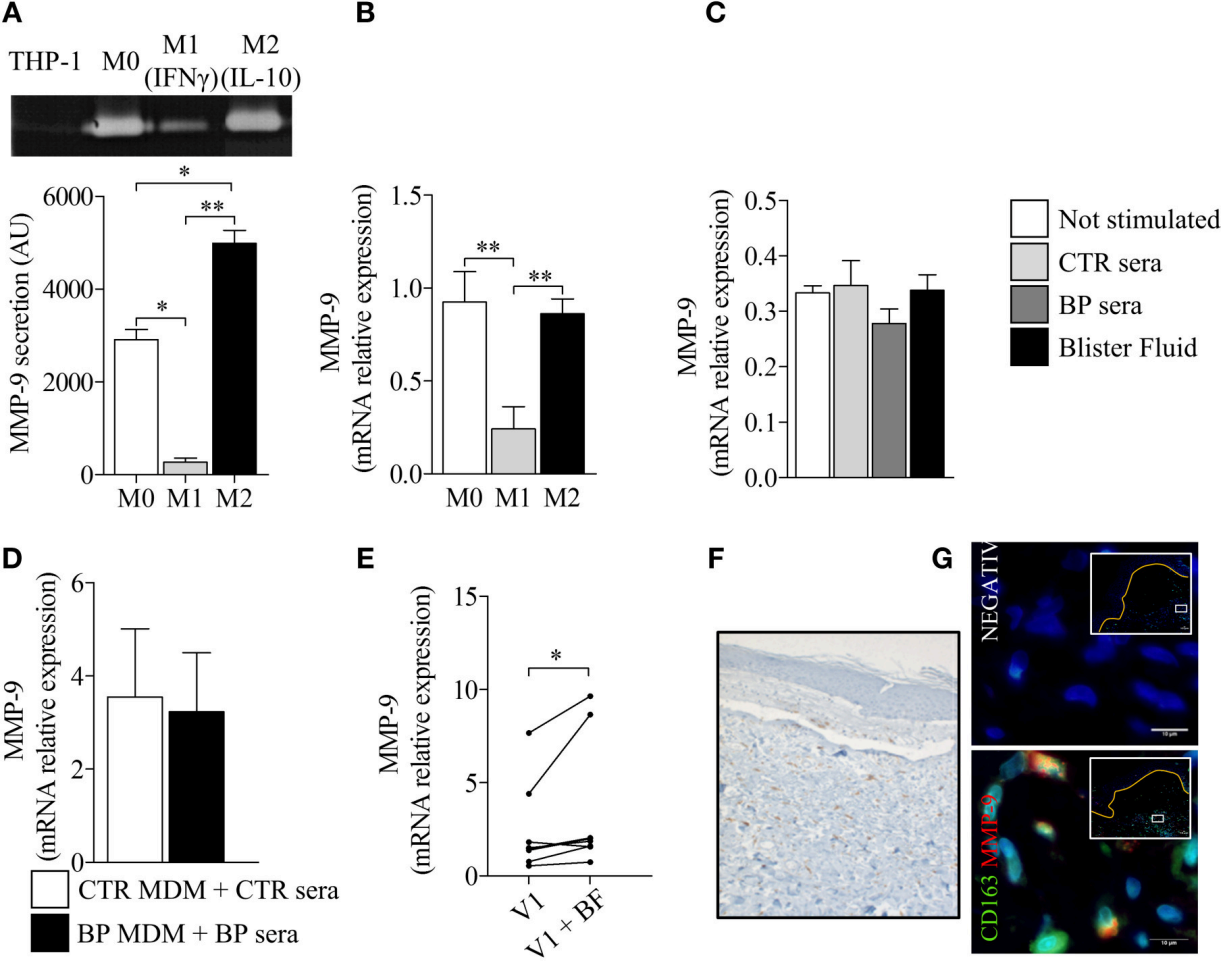
Polarization of BP-MdM influenced their capacity to produce the protease MMP-9. MMP-9 secretion **(A)** and MMP-9 mRNA expression **(B)** were analyzed, respectively, by gelatin zymography and real-time qPCR in M0, M1 (+IFNγ), and M2 (+IL-10) THP-1 derived macrophages. The error bars denote the mean ± SEM. Paired Student's *T*-Test was used for statistics (*n* = 3; ^*^p < 0.05; ^**^p < 0.01). **(C–E)** MMP-9 mRNA expression was analyzed by real-time qPCR in both THP-derived **(C)** and BP monocyte-derived macrophages **(D,E)** stimulated with either control serum, BP serum or blister fluid from BP patient. The error bars denote the mean ± SEM. Nonparametric unpaired Mann-Whitney's test was used for statistical analysis of **(C,D)** (*n* = 8 and *n* = 5, respectively). Nonparametric paired Wilcoxon's test was used to statically analyze **(E)** (*n* = 8; ^*^p < 0.05). **(F,G)** Punch biopsies of lesional skin from patients with BP were subjected to CD163 immuno-staining alone **(F)** or double immunofluorescence staining for MMP-9 (red) and CD163 (green) **(G)** with Hoescht counterstain (blue). Negative CTR, negative control where primary antibodies were not added. White islets show lower magnification images of blister area and orange line designed the dermo-epidermal junction. The boxes in white islets match to the area where the high magnification image has been taken.

To investigate how a M2 type macrophage profile could be associated with disease activity, we analyzed the influence of macrophage polarization on their capacity to produce the protease MMP-9 (Figure 6). To that purpose, PMA-induced THP-1 macrophages were further polarized into M1 and M2 type macrophages with classical stimuli such as IFN-γ and IL-10, respectively. M1 polarization resulted in significant decrease of MMP-9 expression and secretion, whereas this protease was still strongly produced by M2-differentiated THP-1 macrophages (Figures 6A,B). Stimulation with serum and BF also resulted in sustained MMP-9 expression by THP-1 differentiated macrophages (Figure 6C). No differences were noticed between BP serum and control serum on MMP-9 expression both in differentiated THP-1 and in MdM (Figures 6C,D). However, stimulation with BF further increased MMP-9 expression in MdM originated from patients with BP (Figure 6E, *p* < 0.05). Immunohistochemistry studies of skin biopsy specimens of BP revealed a substantial number of CD163^+^ macrophages at lesional site, with double-positive cells CD163^+^ MMP-9^+^ in the blister cavity (Figure 6F).

### Corticosteroids Inhibited the Capacity of M2-macrophages to Produce MMP-9

**Figure 7:**
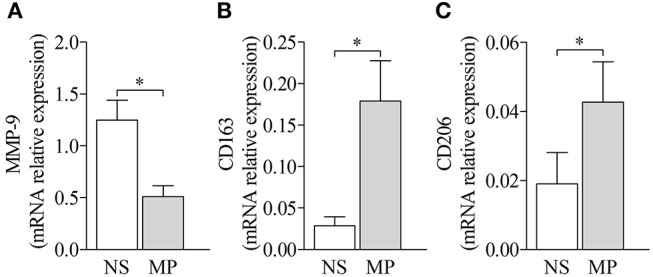
Treatment by methylprednisolone decreased MMP-9 expression and favored M2-type macrophage markers. MMP-9 **(A)** and M2-type macrophage markers CD163 **(B)** and CD206 **(C)** mRNA expressions were analyzed by real-time qPCR in BP MdM treated (MP) or not (NS) with 10 μM methylprednisolone. The error bars denote the mean ± SEM. Nonparametric paired Wilcoxon's test was used (^*^p < 0.05).

Finally, treatment with methylprednisolone reduced MMP-9 expression from BP-derived MdM, while enhancing the expression of both M2 type makers CD163 and CD206 (Figure 7), therefore restoring their tissue regeneration and repair profile.

## Discussion

In this study, we evidenced that macrophage polarization in BP is modified when cells encounter factors present in the BF. Surprisingly, at the site of lesion, macrophages displayed an alternatively activated (M2)-like phenotype and also a high expression level of MMP-9, therefore questioning on the role of macrophages in BP pathophysiology.

Polarization toward M2-type macrophages was dictated at site of lesion in BP. Indeed BFs alone or following serum-induced macrophage differentiation, enhanced the expression of CD163 and CD206 in THP-1-derived macrophages and BP-MdM, respectively, supporting a recent study showing *in situ* the presence of M2-type macrophages in the area of BP skin lesion (6). This is also in line with a polarization toward the M2-phenotype induced by immune complexes and complement components present in the BFs of BP patients (15, 16, 23). It has to be noticed that serum has been shown to influence monocyte maturation and differentiation toward macrophage (24, 25). In line with our recent publication showing that CXCL10 was increased in the blood and in the skin of patients with BP (19), we here demonstrated that serum from BP patients increased CXCL10 expression in THP-1-derived macrophages. However, the increase in CXCL10, but not of the other polarization markers with BP patient serum vs. control serum rather suggests that serum initiates macrophage activation than polarization. Recent works established that THP-1 cells can respond to the polarization protocols used for primary macrophages, and can either match or diverge from what is seen in human peripheral-blood MdMs, then clarifying the discrepancies observed in our study (26, 27). In our study, such differences may be linked to the time of stimulation. Indeed, the THP-1 derived macrophages present the advantages to investigate serum acute effects, whereas patients derived macrophages rather show the steady state reached after long term stimulation. Thus, altogether, our results suggest that in BP macrophage polarization arise in two steps. First macrophages differentiate from monocytes. In BP, those macrophages rather display an activated state than a polarization profile. Then, macrophage M2 polarization occurs when reaching the skin lesional site.

In the initial phase of inflammatory diseases, macrophages are frequently associated with the classically activated M1 phenotype, whereas M2 macrophages are generated in the later phase and are associated with the resolution of inflammation and with the repair process (28). However, in asthma and in allergy, excessive M2 macrophages may increase Th2 cell recruitment, eosinophils infiltration and mucus secretion, and result in airway hyper-responsiveness (13, 29). Our results are of interest as they show that during the active phase of the BP, molecules within the BF orientate macrophages toward an anti-inflammatory M2-like phenotype, which may later influence the skin regeneration process characterized by an absence of scarring in BP. However, this also questioned on the role of macrophages in blister formation. Indeed, it had been previously shown through a mouse BP model that macrophages rather amplified the inflammatory response and blister formation by enhancing neutrophil infiltration in a mast cell-dependent fashion (5).

To conciliate these opposed aspects of the M2-macrophages in BP, we also evaluated the inflammatory status of such polarized macrophages. The pro-inflammatory cytokine TNF-α and IL-10, initially described as immuno-modulatory, were increased in BF-stimulated BP MdMs, in accordance with previous works that showed higher concentrations of both cytokines in BF (30–32). Notably IL-10 displays both pro-and anti-inflammatory effects during immune response, and especially, IL-10 enhances IgE-mediated mast cell responses (33), a pathway which fits with a local pruritic type-2 orientated inflammatory reaction in BP (34). Accordingly we showed that IL-10 concentration was correlated with the activity of the disease, specifically with the inflammatory BPDAI score. In BP, IL-10 may be produced by several cell type of which Th-2 or B-regulatory cells for instances. Then, IL-10 could favor M2-macrophage polarization, and therefore lead to further IL-10 production by M2-macrophages at the skin lesional site. In contrast, IL-23 expression was not induced in BF-stimulated BP MdMs suggesting that the increased level of IL-23 previously observed in the BFs of BP patients (17) cannot be attributed to macrophages. All together these results indicate that M2-type macrophages in BP could increase inflammatory cell recruitment and could participate to the inflammatory cascade observed in BP.

Besides cytokines, elevated levels both of secretion and expression of the metalloproteinase MMP-9 were also associated with M2-type macrophages in BP. Of note, this high level of expression was consecutive to the stimulation with autologous BP serum, and further enhanced in presence of BFs. Noteworthy, by mean of the THP-1 cell line, we reproduced results from previous studies that established a positive correlation between macrophage-released MMP-9 and M2-type polarization (35–37). Indeed, we showed here that macrophage differentiation is already associated with high level of MMP-9, and that M2-macrophage polarization may further enhance MMP-9 production, in contrast to M1 polarization. Therefore, according to the level of MMP-9 observed in BF-stimulated BP MdMs and in setting with the expression of the cytokines described above, we confirm that macrophages rather display a M2-type than a pro-inflammatory M1-type phenotype in BP. Furthermore, such M2-polarization may accentuate the MMP-9 local production initiated by other immune infiltrated cells, and subsequently amplify the local inflammatory process leading to blister formation.

**Figure 8:**
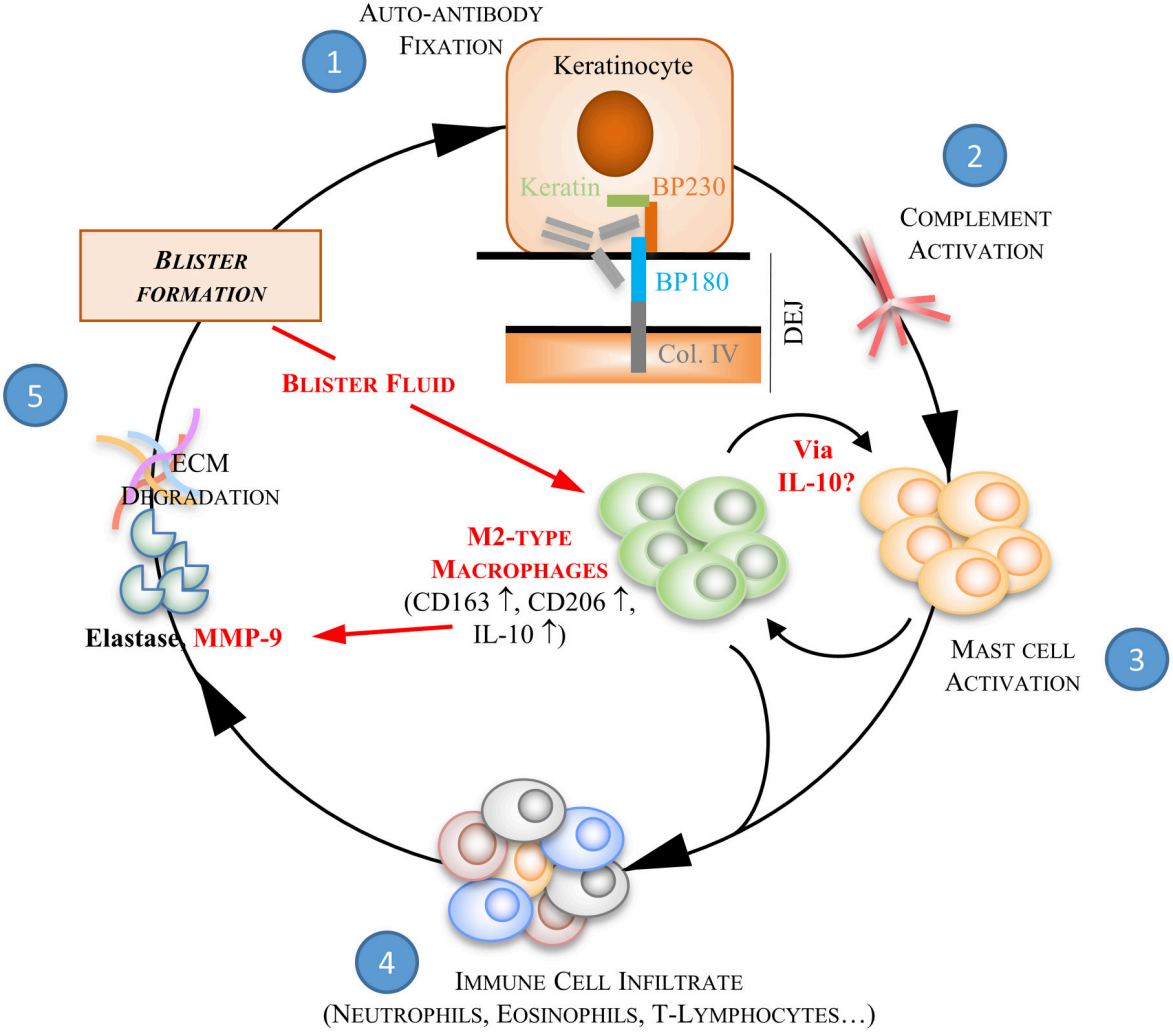
BF-induced M2-type macrophages in the auto-inflammatory response associated to BP. Bullous pemphigoid is characterized by the production of autoantibodies against two proteins of the hemidesmosome structure, BP180 and BP230. The binding of BP autoantibodies ① onto their target initiates the activation of the complement pathway ② which then provokes the release of chemokines and cytokines by mast cells ③. These pro-inflammatory molecules trigger subsequent recruitment of inflammatory cells at the dermal-epidermal junction ④. Inflammatory key mediators secreted by these latter cells induce an overexpression of proteases, such as the matrix-metalloproteinases-9 (MMP9) and the neutrophil elastase, which are involved in extracellular matrix (ECM) degradation and blister formation ⑤. We here further demonstrated the ability of blister fluid to induce M2-type macrophages at site of skin lesion by increasing CD163, CD206, and IL-10 expression. BF-induced M2-type macrophages also show pro-inflammatory function by releasing MMP-9 and then participating to pathophysiological process of BP. Under corticotherapy the auto-amplification process associated to pro-inflammatory cell interaction is dismantled. Indeed, steps ③, ④, and ⑤ are inhibited. In addition, we showed that inhibition of MMP-9 production by methylprednisolone was not associated with a switch toward a M1 macrophage phenotype, but instead reinforced their M2-type phenotype promoting tissue regeneration and repair.

Although the role of macrophages still needs further investigation, our study raises the question of both M2 macrophage polarization and of the associated increase in MMP-9 in the pathophysiological process of BP. Indeed, the protease MMP-9 has been shown as a key molecule for blister formation and any increase in its production is thought to feed the auto-inflammatory and auto-immune processes thereof (7). In contrast, MMP-9 inhibition in patients with BP treated by corticosteroids over a period of 6–9 months, and occasionally even longer, suggests that MMP-9 inhibition does not alter the scarless regeneration process in BP. Furthermore, we here demonstrated that inhibition of MMP-9 production by methylprednisolone was not associated with a switch toward a M1 macrophage phenotype, but instead reinforced their M2-type phenotype promoting tissue regeneration and repair (10, 11) (Figure 8). Then, in the initial inflammatory phase following auto-antibody binding, macrophage recruitment may interact with mast cell to further activate the inflammatory process as previously demonstrated (5). Meanwhile, those macrophages release MMP-9, therefore further increasing the local protease load. Such MMP-9 production may be further elicited by IL-10 produced by other infiltrated and resident immune cells gathered at the skin lesional site, therefore leading to an auto-amplification loop and to disease extent. Under treatment, such pro-inflammatory cell cooperation is inhibited reducing the pro-inflammatory cytokine and proteases release, and subsequently disease activity.

Controlling the amount of MMP-9 locally produced during the active phase of BP disease is clinically of prime importance as MMP-9 along with human elastase are the two main proteases involved in blister formation. In the blood circulation of BP patients, monocytes produce a large amount of MMP-9 (19), which may facilitate their migration into the skin. MMP-9 is secreted in a precursor form (proMMP-9), which must be converted to an active form to exhibit its pathological activity in local lesions. Thus, cytokines such as IL-10 and TNFα may act as a feed forward mechanism creating an auto-amplification loop favoring both M2 polarization and MMP-9 secretion. Other cytokines such as IL-17, CXCL10 released at the site of lesion may further participate to MMP-9 production and activation (17, 19). However, although the use of biologics is of major interest in treatment of autoimmune and auto-inflammatory diseases nowadays, targeting one cytokine or another requires a full understanding of the pathology. Indeed, several case reports highlighted the occurrence of autoimmune bullous skin diseases under anti-TNF therapy (38–42). Based on our results, further preclinical studies are still required to determine whether IL-10 or anti-IL-10 could demonstrate sufficient anti-inflammatory activity in the treatment of BP to warrant further study in a clinical trial.

## Data Availability

All datasets generated for this study are included in the manuscript/supplementary files.

## Author Contributions

PB, FA, and SL designed the study and wrote the manuscript. MR, CM, CB, and SL performed experiments. All authors critically evaluated the data and approved the final version for publication and analyzed clinical and biological data.

### Conflict of Interest Statement

The authors declare that the research was conducted in the absence of any commercial or financial relationships that could be construed as a potential conflict of interest.
